# Unique Biomarker Characteristics in Gestational Diabetes Mellitus Identified by LC-MS-Based Metabolic Profiling

**DOI:** 10.1155/2021/6689414

**Published:** 2021-06-09

**Authors:** Xingjun Meng, Bo Zhu, Yan Liu, Lei Fang, Binbin Yin, Yanni Sun, Mengni Ma, Yuli Huang, Yuning Zhu, Yunlong Zhang

**Affiliations:** ^1^Department of Clinical Laboratory, Women's Hospital, School of Medicine, Zhejiang University, Hangzhou 310006, China; ^2^Institute of Laboratory Medicine, Zhejiang University, Hangzhou 310006, China; ^3^School of Traditional Chinese Medicine, Jinan University, Guangzhou 510632, China; ^4^Department of Cardiology, Shunde Hospital, Southern Medical University (The First People's Hospital of Shunde Foshan), Foshan 528300, China; ^5^Key Laboratory of Neuroscience, School of Basic Medical Sciences, Guangzhou Medical University, Guangzhou 511436, China

## Abstract

**Background:**

Gestational diabetes mellitus (GDM) is a type of glucose intolerance disorder that first occurs during women's pregnancy. The main diagnostic method for GDM is based on the midpregnancy oral glucose tolerance test. The rise of metabolomics has expanded the opportunity to better identify early diagnostic biomarkers and explore possible pathogenesis.

**Methods:**

We collected blood serum from 34 GDM patients and 34 normal controls for a LC-MS-based metabolomics study.

**Results:**

184 metabolites were increased and 86 metabolites were decreased in the positive ion mode, and 65 metabolites were increased and 71 were decreased in the negative ion mode. Also, it was found that the unsaturated fatty acid metabolism was disordered in GDM. Ten metabolites with the most significant differences were selected for follow-up studies. Since the diagnostic specificity and sensitivity of a single differential metabolite are not definitive, we combined these metabolites to prepare a ROC curve. We found a set of metabolite combination with the highest sensitivity and specificity, which included eicosapentaenoic acid, docosahexaenoic acid, docosapentaenoic acid, arachidonic acid, citric acid, *α*-ketoglutaric acid, and genistein. The area under the curves (AUC) value of those metabolites was 0.984 between the GDM and control group.

**Conclusions:**

Our results provide a direction for the mechanism of GDM research and demonstrate the feasibility of developing a diagnostic test that can distinguish between GDM and normal controls clearly. Our findings were helpful to develop novel biomarkers for precision or personalized diagnosis for GDM. In addition, we provide a critical insight into the pathological and biological mechanisms for GDM.

## 1. Introduction

Gestational diabetes mellitus (GDM) is a glucose intolerance disorder that first emerges during women's pregnancy [1]. The prevalence of GDM ranges from 0.6% to 15% across different countries depending on the race and socioeconomic conditions of individuals [2]. It is considered that the increasing incidence of GDM worldwide is due to the growing prevalence of obesity in women of reproductive age and advanced maternal age [3]. GDM poses many health-related concerns with maternal and fetal complications, such as an increased risk for spontaneous preterm birth [4], neonatal hyperbilirubinemia, hypoglycemia [5], shoulder dystocia, stillbirth, acute hospitalization in the neonatal intensive care unit, and respiratory complications [6–9]. More notably, GDM may also lead to a significant increase in long-term incidence of type 2 diabetes and cardiovascular disease in pregnant women [10–18]. In view of these complications, it is important to detect women with GDM as early as possible. In addition, it is critical to utilize and implement current GDM risk-reduction strategies with the aim of minimizing the detrimental gestational complications for mother and offspring. Currently, the accepted GDM diagnostic methods are both time consuming and laborious, which leads to low patient participation. Therefore, it is of utmost importance to find an alternative solution. The advent of advanced technology has made it possible to find more sensitive and specific molecules to distinguish between people with or without GDM. The emergence and development of metabolomics provide deeper insights into the discovery of new biomarkers for metabolic diseases, including GDM. More importantly, metabolomics can offer a noninvasive assessment using maternal biofluids and is a less expensive alternative to other approaches. Based on these advantages, metabolic profiling has the potential to meet the requirement for clinical application and provide critical insight into the pathological and biological mechanisms for GDM. Several studies have already identified early diagnostic markers and explored the pathophysiology of GDM using metabolomics. It has been reported that circulating fatty acids levels, including palmitic acid, stearic acid, and palmitoleic acid, were increased in GDM patients as compared with normal pregnancy groups [19–23]. Additional metabolites were also found to be significantly increased in women with GDM, such as prostanoic acid, sesaminol 2-O-triglucoside, tricin 7-neohesperidoside, dihydro-12-oxo-15-phytoenoic acid [24], acetylcarnitines [21, 22], bile acids [25], ketones [21, 26], creatinine, carbohydrate (primary glucose) [26], and other lipids and organic acids. Reduction of levels in phospholipids, (2E)-14-hydroxytetradec-2-enoic acid (or its isomer), (2E,13R)-13-hydroxytetradec-2-enoic acid, 2,15-dihydroxy-pentadecanoic acid (or its isomers), (7R,8S,9Z,12Z,15Z)-7,8-dihydroxy-9,12,15-octadecatrienoic acid, 11*α*,20,26-trihydroxyecdysone [24], glycerophospholipids [21, 22, 27], 1,5-anhydroglucitol [26], and gluconic acids [21, 27] was also reported. Moreover, decreases in amino acids [21, 22, 27, 28] and fatty acids have been shown [21, 22, 27]. However, some groups have reported that there were no significant changes in metabolite profiles between diagnosed GDM women and healthy controls [29–31]. These discrepancies may be due to differences in sample sizes, variations in study population composition, and statistical methods used.

Considering the promising diagnostic values of metabolomics in GDM, we subjected the serum from 34 normal controls and 34 GDM patients to metabolomics analysis in this study. The main objective of this study is to identify novel biomarkers for precision and personalized diagnosis for GDM and provide a critical insight into the pathological and biological mechanisms for GDM.

## 2. Materials and Methods

### 2.1. Study Population and Sample Collection

Clinical information was collected from 34 pregnant women with GDM and 34 healthy pregnant women with same the gestational weeks who gave birth in Women's Hospital School of Medicine Zhejiang University, Zhejiang, China, between 1 December 2018 and 31 March 2019. Inclusion criteria of pregnant women were as follows: (1) maternal ages at delivery ≥ 20 years; (2) gestational weeks at delivery ≥ 28 weeks; (3) detailed medical records; (4) singleton pregnancy; and (5) no presence of nonhereditary disease.

Exclusion criteria of pregnant women were as follows: (1) multiple pregnancies; (2) stillbirth; (3) in vitro fertilization-embryo transfer; and (4) had chronic diseases. Participants' blood samples were venously collected after 8-14 hours of fasting during their second trimester of pregnancy (24–28 gestational weeks). Sample transfer centrifugation (3500 rpm for 10 min at 4°C) and separation of serum were completed within 1 hour. Final samples were stored at -80°C until retrieval for metabolomics analysis.

### 2.2. The Diagnostic Criteria of GDM

The diagnosis of GDM cases were identified using the oral glucose tolerance test (OGTT) conducted between 24 and 28 gestational weeks. According to the International Association of Diabetes and Pregnancy Study Groups (IADPSG) criteria [32], pregnant women were considered to have GDM if one of the following plasma glucose values was met or exceeded: 0 h, 5.1 mmol/L; 1 h, 10.0 mmol/L; or 2 h, 8.5 mmol/L, after a 75 g glucose load.

## 3. Metabolomics Analysis

### 3.1. Metabolites Extraction

The serum samples had been thawed once prior to use for our study. The serum samples (100 *μ*l) were resuspended with prechilled 80% methanol and 0.1% formic acid and vortex oscillation mix. Samples were placed in an ice bath for 5 min and then centrifuged at 15,000 rpm, 4°C for 10 min. A calculated amount of supernatant was diluted to a final concentration containing 60% methanol by LC-MS grade water and subsequently transferred to a fresh Eppendorf tube with 0.22 *μ*m filter (Millipore, Bedford, MA, USA). Samples were then centrifugated at 15,000 g, 4°C for 10 min. Finally, the filtrate was injected into the LC-MS/MS system for analysis. Equal volume samples from each experimental sample were mixed as quality control (QC) samples. Blank sample is the blank matrix of the experimental sample, and the pretreatment process of the sample is the same as that of the experimental sample.

### 3.2. LC-MS Analysis (Liquid Chromatography-Mass Spectrometry)

LC-MS/MS analyses were performed using a Vanquish UHPLC system (Thermo Fisher Scientific, Waltham, MA, USA) coupled with an Orbitrap Q Exactive series mass spectrometer (Thermo Fisher Scientific, Waltham, MA, USA). Samples were injected into a Hypersil Gold column (C18) (Thermo Fisher Scientific, Waltham, MA, USA) using a 16 min linear gradient at a flow rate of 0.2 ml/min. The eluents for the positive polarity mode were eluent A (0.1% formic acid in water) and eluent B (methanol). The eluents for the negative polarity mode were eluent A (5 mM ammonium acetate, pH 9.0) and eluent B (methanol). The solvent gradient was set as follows: 2% B, 1.5 min; 100% B, 12.0 min; 100% B, 14.0 min; 2% B, 14.1 min; and 2% B, 16 min. The Q Exactive mass series spectrometer was operated in positive/negative polarity mode with spray voltage of 3.2 kV, capillary temperature of 320°C, sheath gas flow rate of 35 arb, and aux gas flow rate of 10 arb.

### 3.3. Identification of Metabolites

The raw data files generated by UHPLC-MS/MS were processed using the Compound Discoverer 3.0 (CD3.0, Thermo Fisher Scientific, Waltham, MA, USA) to perform peak alignment, peak picking, and quantitation for each metabolite. The main parameters were set as follows: retention time deviation of 0.1 min; quality deviation of 5 ppm; signal strength deviation of 30%; signal-to-noise ratio of 3; and minimum signal strength of 100,000. Peak intensities were normalized to the total spectral intensity. The normalized data was then used to predict the molecular formula based on additive ions, molecular ion peaks, and fragment ions. Lastly, peaks were matched with the mzCloud (https://www.mzcloud.org/) and ChemSpider (http://www.chemspider.com/) databases to obtain the accurate qualitative and relative quantitative results.

## 4. Data Analysis

After the serum metabolites assessment, the metabolites were annotated using the Kyoto Encyclopedia of Genes and Genomes (KEGG) database (http://www.genome.jp/kegg/), Human Metabolome Database (HMDB) database (http://www.hmdb.ca/), and Lipidmaps database (http://www.lipidmaps.org/). Principal components analysis (PCA), partial least squares discriminant analysis (PLS-DA), and fold change (FC) analysis were performed at metaX (a flexible and comprehensive software for processing metabolomics data). A univariate analysis (*t*-test) was applied to calculate the statistical significance (*p*-value). In this study, the Bonferroni method was used to reduce the false discovery rate (FDR). The metabolites with variable importance in the projection (VIP) > 1, *p* − value < 0.05, and fold change ≥ 2 or FC ≤ 0.5 were considered differential metabolites. Volcano plots were used to filter metabolites of interest, which were based on Log_2_(FC) and −Log_10_(*p* − value) of metabolites.

The data used for clustering heat maps was normalized using *z*-scores of the intensity areas of differential metabolites and was plotted by the Pheatmap package in *R* language (version 3.5.1). The correlation between differential metabolites was analyzed by *cor()* in *R* language (method: *Pearson*). Statistically significant values of correlation between differential metabolites were calculated by *cor.mtest* in *R* language. *p* − value < 0.05 was considered statistically significant, and correlation plots were plotted by *corrplot* package in *R* language. The functions of these metabolites and metabolic pathways were studied using the KEGG database. The metabolic pathway enrichment of differential metabolites was performed when ratio was satisfied by *x*/*n* > *y*/*N*. Metabolic pathway enrichments were considered statistically significant when *p*-value of metabolic pathway is <0.05.

## 5. Statistical Analysis

The SPSS software version 20 (SPSS Inc., Chicago, IL, USA) was used for statistical analysis and receiver operating characteristic (ROC) curve preparation. The modeling methods were selected on the basis of the logistic regression to increase the diagnostic accuracy. When the data was not normally distributed, normal transformations were attempted using of area normalization method. For the data processing portion, Log_2_ conversion and standardization was completed; then, we performed UV scale and a two-tailed *t*-test to calculate the *p*-value. In this study, we used the Log_2_ conversion to make the data meet the normality of the distribution. Chi-square test was used for categorical data and the Student's **t**-test for measurement data between two groups. All collected data was expressed as the mean ± standard error of the mean (SEM), and the statistical significance level was set at *p* < 0.05.

## 6. Results

### 6.1. Comparison of Clinical Data

A total of 68 individuals, 34 normal pregnant women, and 34 pregnant women with GDM were included in this study. Detailed clinical data were recorded for all participants. The mean maternal age of two groups was 28.35 ± 3.03 and 31.78 ± 4.61 years, respectively. A comparison of height, weight, age for marriage, gravidity, systolic blood pressure, diastolic blood pressure, weight gain, alanine aminotransferase (ALT), and aspartate aminotransferase (AST) was listed in Table 1. In addition, other laboratory data (Table S1) showed no significant differences between the GDM and control groups. Body mass index (BMI), fasting glucose and insulin, 1 h glucose, 2 h glucose and insulin, hemoglobin A1 (HbA1C), homeostasis model of assessment-insulin resistance (HOMA-IR), and triacylglycerides (TG) were significantly higher in the GDM group than in the control group (*p* < 0.05, Table 1). Gestational weeks and high-density lipoprotein- (HDL-) cholesterol were significantly lower in the GDM group compared with the control group (*p* < 0.05, Table 1). No differences between the two groups (*p* > 0.05, Table 1) were observed in the following parameters: age for marriage, height, weight, weight gain, gravidity, parity, systolic blood pressure, diastolic blood pressure, 1 h insulin, and total cholesterol (TCH).

### 6.2. Metabolic Results

To maximize the identification of different metabolites, we tested the samples in both the positive ion mode and negative ion mode. QC samples were included to determine the state of the instrument and to evaluate the stability of the system during the whole experiment. The correlations of QC samples were all close to 1, indicating that the method used has high stability and good data quality (Figures S2(a) and S2 (b)). The peak obtained from all experimental samples and QC samples were extracted and Pareto scaling was applied for PCA analysis. In the PCA analysis diagram, the distribution of QC samples, GDM samples, and control samples is clustered together. These results further indicate that the model we employed is reliable (Figures S2(c)–S2(f)).

**Figure 1:**
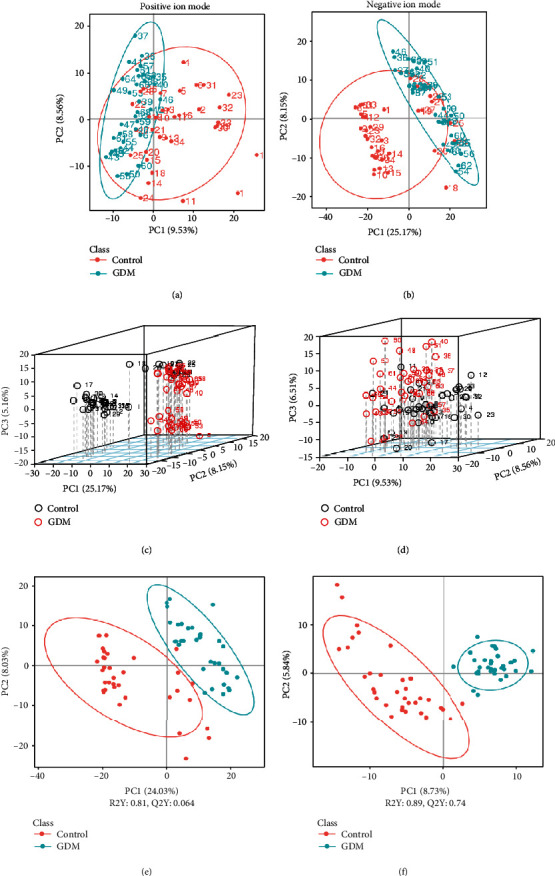
Metabolomic analysis of control and GDM. (a, b) PCA analysis between control and GDM. (c, d) 3D score plot of PCA analysis between control and GDM. (e, f) PLS-DA analysis between control and GDM, (a, c, e) Positive ion mode. (b, d, f) Negative ion mode.

Next, we analyzed the differential metabolites. PCA analysis was used as an unsupervised method, and PLS-DA was used as a supervised method to get an overview of the data and to detect trends within the experiment. A clear separation can be observed between GDM and control groups from the LC-MS data, indicating metabolic changes are inherent to GDM (Figures 1(a)–1(f)).

**Figure 2:**
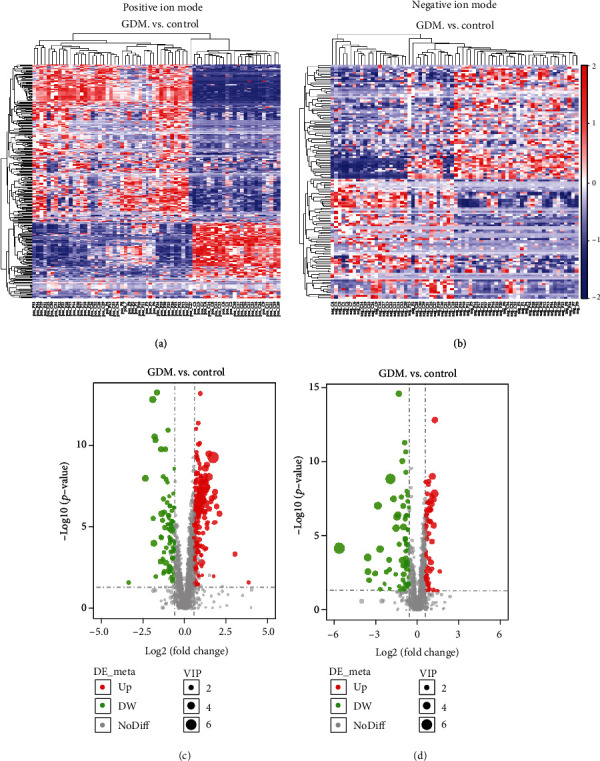
Analysis of differential metabolites between control and GDM through LC-MS. (a, b) Hierarchical clustering analysis was performed on each group of differential metabolites obtained, and the relative quantitative values of the differential metabolites were converted into *z* values (*z* = (*x*–*μ*)/*σ*: *x* is a specific fraction, *μ* is average Number, and *σ* is the standard deviation) and clustering; different color regions represent different clustering group information, similar to the metabolic expression patterns in the same group, and may have similar functions or participate in the same biological process. (c, d) For each metabolite difference multiple, take the logarithm of 2 as the base, and take the *p*-value to the absolute value of the logarithm of 10 to make the volcano map. (a, c) Positive ion mode. (b, d) Negative ion mode.

Based on the compounds identified by LC-MS, we generated a metabolite heat map and volcano map that revealed considerable differences between healthy controls and pregnant women with GDM (Figure 2). It can be seen that under the positive ion mode, a total of 2022 metabolites were detected with 184 increased metabolites and 86 decreased metabolites (Figures 2(a) and 2(c)). In the negative ion mode, a total of 1299 metabolites were detected with 65 increased metabolites and 71 decreased metabolites (Figures 2(b) and 2(d)). To better identify the potential biomarkers for GDM, we selected the top 40 differential changed metabolites between GDM patients and the normal pregnancy controls (20 for positive ion mode, and 20 for negative ion modes, respectively) (Table S3).

**Figure 3:**
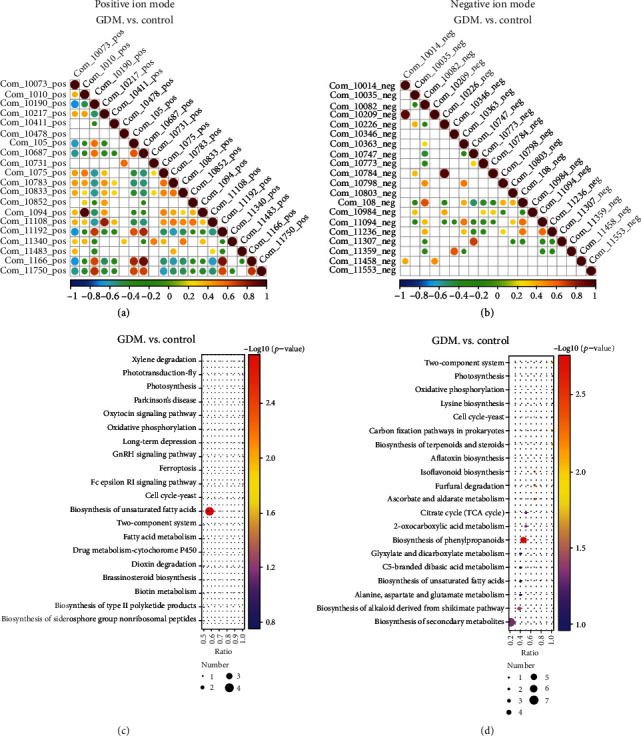
Differential metabolite KEGG pathway enrichment map. (a, b) When the linear relationship of the two metabolites is enhanced, the correlation coefficient tends to 1 or -1; when positive correlation, it tends to 1, and when it is negatively correlated, it tends to -1. The correlation is a maximum of 1, a complete positive correlation (red), a correlation of -1, and a complete negative correlation (blue). (c, d) KEGG analysis was used to identify the pathways which were significantly enriched by differential metabolites compared to all identified metabolite backgrounds. (a, c) Positive ion mode. (b, d) Negative ion mode.

Next, correlation analysis of differential metabolites and KEGG pathway enrichment prediction were performed, and a KEGG enrichment bubble map was generated. The biosynthesis of unsaturated fatty acids, biosynthesis of phenylpropanoids, carbon fixation pathways in prokaryotes, biosynthesis of terpenoids and steroids, two-component system, ascorbate and aldarate metabolism, furfural degradation, isoflavonoid biosynthesis, biosynthesis of alkaloids derived from shikimate pathway, and biosynthesis of secondary metabolites were all found to be statistically different between the control group and the GDM group (Figures 3(a)–3(d); Table S4). In these pathways, docosahexaenoic acid, docosapentaenoic acid, arachidonic acid, citric acid, *α*-ketoglutaric acid, phosphoric acid, dehydroascorbic acid, 2-furoic acid, cephaeline, and methyl jasmonate were upregulated, and isoliquiritigenin, genistein, daidzein, and typhasterol were downregulated. In the positive ion mode, the only pathway with statistical differences was the biosynthesis of unsaturated fatty acids (Table S4). From these results, we selected the differential metabolites in this pathway for further analysis: eicosapentaenoic acid, docosahexaenoic acid, docosapentaenoic acid, and arachidonic acid. In the negative ion mode, differential metabolites were selected based on the following principles: (1) VIP values > 3; (2) KEGG metabolic pathway; and (3) 20 metabolites with the smallest *p*-value (Tables S4 and S5). The overlapping metabolites that fell within criteria of these three conditions were selected for subsequent analysis. These metabolites include citric acid, *α*-ketoglutaric acid, genistein, daidzein, phosphoric acid, and 2-furoic acid, which can be screened in both positive ion mode and negative ion modes. The FC, VIP, and AUC of these ten metabolites are listed in Table 2.

### 6.3. Validation and Diagnostic Performance of Selected Metabolite

**Figure 4:**
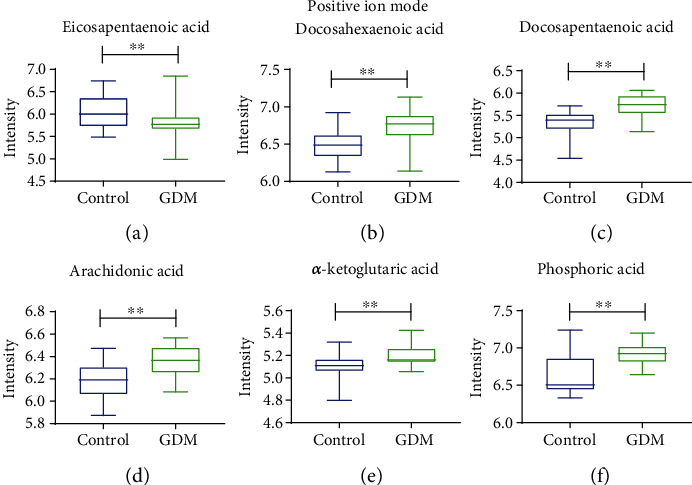
Boxplots of metabolites between control and GDM under positive ion mode. Student's *t*-test for measurement data between two groups was performed for significant difference. All data are expressed as the mean ± SEM, and the statistical significance level was set at ^∗^*p* < 0.05. ^∗∗^*p* < 0.01.

**Figure 5:**
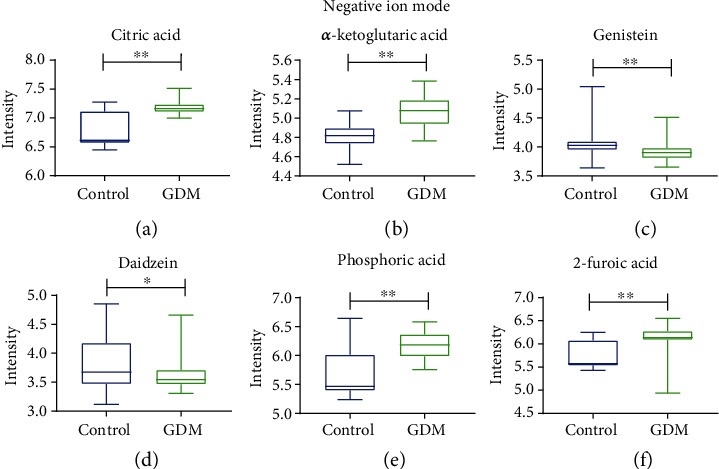
Boxplots of metabolites between control and GDM under negative ion mode. Student's *t*-test for measurement data between two groups was performed for significant difference. All data are expressed as the mean ± SEM, and the statistical significance level was set at ^∗^*p* < 0.05. ^∗∗^*p* < 0.01.

The levels of the selected metabolites in a group of participants comprising of 34 normal women and 34 women with GDM were measured using LC-MS and analyzed by the Student's *t*-test. Figures 4 and 5 depict the boxplots of their concentration levels in the GDM and control.  Table 3 shows the group AUC values of targeted metabolites obtained through multiple comparison analysis. The AUC values indicate the diagnostic potentials of the metabolites as unique biomarkers for identification of GDM and control.

The area under the curve for the individual differential metabolites we selected was less than 0.882 (Table 2). However, we hope to find a combination of metabolites which has higher sensitivity and specific to distinguish between GDM and controls. We decided to combine the metabolites into various sets and subjected them to AUC analysis to evaluate their diagnostic performances as combined biomarkers for GDM. Based on the principle of selecting the least metabolites and the highest area under the curve, we have selected the following combination: eicosapentaenoic acid, docosahexaenoic acid, docosapentaenoic acid, arachidonic acid, citric acid, *α*-ketoglutaric acid, and genistein. The AUC value of the combined metabolites was 0.984 between the GDM and control groups (Table 3; Figure S6).

## 7. Discussion

Alteration in metabolites, like bile acid metabolism, amino acid metabolism, and fatty acid metabolism, has all been involved with the development of metabolic diseases and is characterized as a hallmark of metabolic diseases, such as GDM [31–33]. The emergence and development of metabolomics provide deeper insights in the discovery of new biomarkers of these diseases [34]. More importantly, metabolomics can offer a noninvasive assessment by using maternal biofluids and is a less expensive alternative to other approaches [35]. GDM can be diagnosed using the OGTT method, which is a cheap “golden diagnostic standard”. Unfortunately, it may be not the ideal biomarker to predict the potential mechanism related to GDM [36]. In this study, we identified several differential metabolites, such as eicosapentaenoic acid, docosahexaenoic acid, docosapentaenoic acid, and arachidonic acid, that were closely correlated to the disease process of GDM. Thus, the metabolomic biomarkers have the potential to serve as an innovative approach for the predictive, preventive, and personalized medicine in the future.

Our analysis of clinical data shows that triacylglycerides were significantly increased in the GDM group, while HDL-cholesterol and low-density lipoprotein- (LDL-) cholesterol decreased significantly in the GDM group (Table 2). This finding suggests there is a change in lipid metabolism during women's pregnancy. Our study identified four molecules in the pathway of unsaturated fatty acid metabolism, which includes eicosapentaenoic acid, docosahexaenoic acid, docosapentaenoic acid, and arachidonic acid.

Pregnancy is a complicated physiological process, and pregnant women need sufficient nutrition to support the growth and development of themselves and their fetus. It has been found that arachidonic acid (AA) and docosahexaenoic acid (DHA) play important roles in fetal growth and development [37, 38]. However, these enzyme expressions to synthesize long-chain polyunsaturated fatty acids (LC-PUFA) are quite low in the fetus. This shows that AA and DHA, which is necessary for fetal growth and development, are supplied by the placental transport [39]. Therefore, alterations in maternal polyunsaturated fatty acid (PUFA) metabolism during gestation would significantly impact the growth and development of the fetus. In addition, research has been reported that in placental transfer of AA in vitro in perfused placentas of women with insulin-dependent diabetes mellitus was impaired [40].

Williams et al. found that linoleic acid, oleic acid, myristic acid, D-galactose, D-sorbitol, O-phosphocolamine, L-alanine, L-valine, 5-hydroxy-l-tryptophan, L-serine, sarcosine, L-pyroglutamic acid, L-mimosine, L-lactic acid, glycolic acid, fumaric acid, and urea differentiated GDM cases from controls using GC-MS technology in early pregnancy and identified combinations of metabolites in early pregnancy that are associated with subsequent risk of GDM [41].

In this study, we also found some additional branched-chain amino acids that can be used to distinguish GDM from the control group using untargeted metabolomics. It was found that DL-*β*-leucine, L-threonine, L-(+)-alanine, DL-serine, valine, L-tyrosine, *α*-linolenic acid, oleic acid, tryptophan, and glutamine have no significant change between controls and GDM; L-isoleucine, L-theanine, L-aspartic acid, L-phenylalanine, cystine, L-glutamic acid, and DL-lysine have significant changes. Among the 17 metabolites discovered by Williams et al., most of them were not detected in our study. Based on these results, it is reasonable to infer that metabolism is different in the early and middle trimesters of pregnancy.

Although we find *α*-linolenic acid (ALA) has no significant change between controls and GDM in our study, our results show that DHA was increased in GDM. DHA can be converted from ALA [42, 43]. Circulating levels of DHA can reflect the ability of synthesize by the liver and the amount of dietary intake. Previously, White et al. performed to compare obese women with GDM with obese non-GDM women at time point 1 (mean gestational weeks 17 weeks 0 days) and time point 2 (mean gestational weeks 27 weeks 5 days) using a targeted NMR metabolome [44]. The results showed that total fatty acids were higher at time point 1 and were marginally increased at time point 2. Additionally, monounsaturated fatty acid and saturated fatty acid concentrations were greater at both time points. At time point 2, a decreased proportion of DHA and increased proportion of saturated fatty acids both reached significance in GDM women. On the contrary, our results show that DHA was increased in GDM. The difference in DHA findings may be attributed to the choice of different groups of people. Furthermore, White et al.'s research found that tricarboxylic acid (TCA) cycle intermediate citrate concentrations were also higher but lactate had no notable difference between the two groups at either time [44]. This is basically consistent with our findings that citric acid was increased in GDM, while ethyl lactate and n-butyl lactate do not change significantly.

Findings from metabolomics of GDM have generally been inconsistent in the past. Alterations in branched-chain amino acids, free fatty acids, fatty acid oxidation products, and gluconeogenic precursors have been reported by several studies [41, 45–47]. Yet, Graça et al. and Sachse et al. found no significant changes in metabolite profiles between women with GDM and controls [30, 31]. Surprisingly, our results are not consistent with the findings of others. The reasons for the different results may be as follows: utilization of metabolome profiling platforms, differences in specimen collections, GDM diagnostic criteria, intrinsic biological characteristics of individual participants, methods in data processing, and statistical analysis. The implementation of strict study guidelines and consistent recommendations across studies are needed to improve replication of findings [48].

Based on our results, we can see that under the positive ion mode, a total of 2022 metabolites were detected and a total of 1299 metabolites were detected in the negative ion mode. Through more rigorous selection, we selected the most obvious metabolites to prepare an ROC curve. Moreover, the results of eicosapentaenoic acids, docosahexaenoic acid, docosapentaenoic acid, and arachidonic acid are inconsistent [49–51]. Wheeler's experimental results are relatively similar to ours; they found that eicosapentaenoic acid, docosahexaenoic acid, docosapentaenoic acid, and arachidonic acid were all upregulated [23]. The only difference in our study is that eicosapentaenoic acid was reduced. Also, his research found another furan fatty acid metabolite 3-carboxy-4-methyl-5-propyl-2-furanpropanoic acid (CMPF) can impair *β* cell function by inhibiting insulin biosynthesis and secretion through organic anion transporters-3 (OAT3). This can cause abnormal glucose metabolism and increased oxidative stress [23]. This may be further explained by glucolipotoxicity, which means hyperglycemia and hyperlipidemia arise and may exert additional damaging effects on the *β* cell. Many studies have associated glucolipotoxicity with *β* cell dysfunction in type 2 diabetes, suggesting that metabolites are likely causally related to diabetes development [52]. This proposition may offer some insight on the results of Wheeler's and ours. Fatty acids have been proven to induce *β* cell apoptosis under high glucose conditions [53]. Pancreatic *β* cells exposed to fatty acids for a long period can lead to increased oxidative stress products like ROS. High levels of ROS can increase cell membrane permeability through oxidation of lipid, leading to calcium influx, and phospholipase activation, which may further induce *β* cell apoptosis [54]. Busch et al. have also found that the expression of the enzyme, stearoyl coenzyme A desaturase, correlates with the resistance of *β* cells to the proapoptotic by the effect of palmitate. This may indicate that the capability of cell to desaturate fatty acids serves as some form of protection against glucolipotoxicity [55]. These results suggest that unsaturated fatty acids are at least partially involved in the development of GDM. Under normal physiological conditions, eicosapentaenoic acid, docosahexaenoic acid, and arachidonic acid can be synthesized from essential fatty acid precursors that might otherwise be inadequate during periods of rapid intrauterine growth, which are considered essential for intrauterine development and specific functions such as retinal and cerebral development [38, 56–58].

Ultimately, we want to develop an effective discriminant model based on a ten-metabolite panel that can predict GDM early. We screened only one pathway in positive ion mode where eicosapentaenoic acid, docosahexaenoic acid, docosapentaenoic acid, and arachidonic acid were included in this pathway. We combined those four molecules with other metabolites into various sets and subjected them to AUC analysis in order to evaluate their diagnostic performances as combined biomarkers for controls and GDM. Based on the principle of selecting the least metabolites and the highest area under the curve, we have selected the following combinations: eicosapentaenoic acid, docosahexaenoic acid, docosapentaenoic acid, arachidonic acid, citric acid, *α*-ketoglutaric acid, and genistein. The AUC value of those metabolites was 0.984 between the GDM and control groups. This result shows that these metabolite combinations can clearly distinguish between GDM and normal controls. However, this would require a larger sample size for subsequent verification. In continuation from our initial findings, we will work on verifying these results with hopes of using them clinically in the near future.

Overall, our study had several limitations. First, the number of patients included might not be of a substantial amount and could have affected the robustness of our statistical analysis. Therefore, the conclusions of this study need to be verified using a larger group of participants. Another limitation of our study is that some external factors outside of the controlled screening parameters may have affected the metabolome outcomes. These factors include some lifestyle elements, such as dietary habits, previous macrosomia, and previous GDM. Similarly, glycemic control for all women included in the present study was not able to be characterized because these data was not available. Lastly, the LC-MS analysis we performed might not be a feasible screening technique for large populations because of its high cost. In this study, we found several differential factors, such as gestational weeks and BMI, and these factors may be related to the differences in metabolites. As a metabolic disease, GDM may be related to the dysfunctional lipid metabolism, obesity, and glucose metabolism. In this study, we mainly focus on the differential metabolites between the controls and GDM groups, and these metabolites may be the candidates or biomarkers for GDM diagnosis. We will further our studies to explore the potential mechanism and correlation between these differential metabolites and other factors in the future.

In this study, we found that unsaturated fatty acid metabolism was impaired in GDM by identifying key metabolites differences between the controls and GDM groups. In addition, we discovered a set of metabolite combination that can clearly distinguish between GDM and normal controls. These results demonstrate the possibility of developing a diagnostic test that can distinguish between GDM and normal controls clearly.

## Figures and Tables

**Table 1:** General characteristics of study subjects.

Characteristic	No GDM	GDM	*p*-value
Age, years	28.35 ± 3.03	31.78 ± 4.61	0.001
Age for marriage, years	26.12 ± 2.56	27.2 ± 3.04	0.115
Height, cm	161 ± 5.26	159 ± 4.67	0.138
Weight, kg	57.85 ± 6.36	62.13 ± 7.77	0.016
BMI, kg/m^2^	22.33 ± 2.6	24.45 ± 2.45	0.001
Weight gain, kg	6.26 ± 2.83	6.33 ± 3.91	0.930
BMI before pregnant, kg/m^2^	19.91 ± 2.3	21.96 ± 2.4	0.001
Gravidity	1.53 ± 0.75	1.88 ± 1.00	0.149
Parity			
Nulliparous	22	16	
Multiparous	12	18	0.147
Gestational weeks	39.24 ± 1.07	38.44 ± 1.30	0.008
Systolic blood pressure (mm Hg)	113.6 ± 9.13	118.3 ± 10.6	0.055
Diastolic blood pressure (mm Hg)	66.6 ± 8.9	68.5 ± 10.4	0.420
Fasting glucose (mmol/l)	4.37 ± 0.34	5.07 ± 0.68	*p* ≤ 0.001
1 h glucose, OGTT (mmol/l)	7.44 ± 1.21	10.70 ± 1.33	*p* ≤ 0.001
2 h glucose, OGTT (mmol/l)	6.32 ± 0.75	9.43 ± 1.51	*p* ≤ 0.001
HbA1C (%)	4.8 ± 0.21	5.3 ± 0.39	*p* ≤ 0.001
Insulin (*μ*U/ml)	7.21 ± 2.95	10.29 ± 4.35	*p* ≤ 0.001
1 h insulin (*μ*U/ml)	61.69 ± 24.55	68.73 ± 38.49	0.372
2 h insulin (*μ*U/ml)	54.12 ± 25.81	86.26 ± 47.29	0.001
HOMA-IR	1.42 ± 0.64	2.33 ± 0.18	*p* ≤ 0.001
Triacylglycerides (mmol/l)	1.95 ± 0.57	2.86 ± 1.44	0.001
Total cholesterol (mmol/l)	6.04 ± 0.79	5.65 ± 1.05	0.083
LDL-cholesterol (mmol/l)	3.08 ± 0.63	2.63 ± 0.70	0.007
HDL-cholesterol (mmol/l)	2.09 ± 0.42	1.78 ± 0.41	0.003
ALT, median(range), U/l	20.29 ± 9.43	18.26 ± 14.08	0.500
AST, median(range), U/l	19.71 ± 5.33	17.65 ± 7.28	0.188

GDM, gestational diabetes mellitus; HbA1c, hemoglobin A1; HOMA-IR, homeostasis model of assessment-insulin resistance; ALT, alanine aminotransferase; AST, aspartate aminotransferase. Data are mean ± SEM. *t*-test in continuous variables and chi-square test in categorical data were performed as appropriate. Results were considered significant when *p* < 0.05, compared with control group.

**Table 2:** Ten major differential metabolites for future analysis.

ID	Name_des	Formula	Molecular weight	RT (min)	FC	*p*-value	VIP	Up/down
*ESI+*								
Com_962_pos	Eicosapentaenoic acid	C20 H30 O2	302.22	14.10	0.58	4.95E-03	2.28	Down
Com_384_pos	Docosahexaenoic acid	C22 H32 O2	328.24	15.07	1.77	1.62E-06	1.60	Up
Com_2412_pos	Docosapentaenoic acid	C22 H34 O2	330.26	15.24	2.36	6.97E-09	2.23	Up
Com_1075_pos	Arachidonic acid	C20 H32 O2	304.24	15.13	1.50	3.85E-06	1.06	Up
Com_7642_pos	*α*-Ketoglutaric acid	C5 H6 O5	146.02	1.40	1.18	1.22E-03	0.44	Up
Com_332_pos	Phosphoric acid	H3 O4 P	97.98	1.68	1.65	9.31E-07	1.80	Up
*ESI-*								
Com_108_neg	Citric acid	C6 H8 O7	192.03	1.49	2.11	1.02E-09	3.43	Up
Com_5332_neg	*α*-Ketoglutaric acid	C5 H6 O5	146.02	1.72	1.78	2.17E-09	2.00	Up
Com_7586_neg	Genistein	C15 H10 O5	270.05	9.61	0.63	6.15E-03	1.17	Down
Com_8251_neg	Daidzein	C15 H10 O4	254.06	9.10	0.52	4.12E-02	1.50	Down
Com_740_neg	Phosphoric acid	H3 O4 P	97.98	1.55	2.33	1.42E-08	4.31	Up
Com_783_neg	2-Furoic acid	C5 H4 O3	112.02	1.50	2.25	1.97E-06	3.15	Up

FC, fold change (GDM case/control). VIP, variable importance in the projection. ESI+, positive ion mode; ESI-, negative ion mode.

**Table 3:** Area under the curves among GDM and control.

Group	AUC	95% CIs
Eicosapentaenoic acid; docosahexaenoic acid; docosapentaenoic acid; arachidonic acid	0.909	0.840-0.979
Eicosapentaenoic acid; docosahexaenoic acid; docosapentaenoic acid; arachidonic acid; citric acid	0.959	0.921-0.998
Eicosapentaenoic acid; docosahexaenoic acid; docosapentaenoic acid; arachidonic acid; *α*-ketoglutaric acid	0.981	0.957-1.000
Eicosapentaenoic acid; docosahexaenoic acid; docosapentaenoic acid; arachidonic acid; genistein	0.913	0.846-0.981
Eicosapentaenoic acid; docosahexaenoic acid; docosapentaenoic acid; arachidonic acid; daidzein	0.920	0.858-0.983
Eicosapentaenoic acid; docosahexaenoic acid; docosapentaenoic acid; arachidonic acid; phosphoric acid	0.952	0.906-0.997
Eicosapentaenoic acid; docosahexaenoic acid; docosapentaenoic acid; arachidonic acid; 2-furoic acid	0.957	0.916-0.997
Eicosapentaenoic acid; docosahexaenoic acid; docosapentaenoic acid; arachidonic acid; citric acid; *α*-ketoglutaric acid	0.984	0.962-1.000
Eicosapentaenoic acid; docosahexaenoic acid; docosapentaenoic acid; arachidonic acid; citric acid; genistein	0.960	0.922-0.998
Eicosapentaenoic acid; docosahexaenoic acid; docosapentaenoic acid; arachidonic acid; citric acid; daidzein	0.958	0.918-0.997
Eicosapentaenoic acid; docosahexaenoic acid; docosapentaenoic acid; arachidonic acid; citric acid; phosphoric acid	0.958	0.917-0.998
Eicosapentaenoic acid; docosahexaenoic acid; docosapentaenoic acid; arachidonic acid; citric acid, 2-furoic acid	0.960	0.922-0.998
Eicosapentaenoic acid; docosahexaenoic acid; docosapentaenoic acid; arachidonic acid; *α*-ketoglutaric acid; genistein	0.982	0.959-1.000
Eicosapentaenoic acid; docosahexaenoic acid; docosapentaenoic acid; arachidonic acid; *α*-ketoglutaric acid; daidzein	0.981	0.957-1.000
Eicosapentaenoic acid; docosahexaenoic acid; docosapentaenoic acid; arachidonic acid; *α*-ketoglutaric acid; phosphoric acid	0.982	0.959-1.000
Eicosapentaenoic acid; docosahexaenoic acid; docosapentaenoic acid; arachidonic acid; *α*-ketoglutaric acid; 2-furoic acid	0.981	0.957-1.000
Eicosapentaenoic acid; docosahexaenoic acid; docosapentaenoic acid; arachidonic acid; genistein; daidzein	0.922	0.860-0.984
Eicosapentaenoic acid; docosahexaenoic acid; docosapentaenoic acid; arachidonic acid; genistein; phosphoric acid	0.951	0.905-0.997
Eicosapentaenoic acid; docosahexaenoic acid; docosapentaenoic acid; arachidonic acid; genistein; 2-furoic acid	0.958	0.919-0.998
Eicosapentaenoic acid; docosahexaenoic acid; docosapentaenoic acid; arachidonic acid; daidzein; phosphoric acid	0.956	0.913-0.999
Eicosapentaenoic acid; docosahexaenoic acid; docosapentaenoic acid; arachidonic acid; daidzein; 2-furoic acid	0.956	0.915-0.997
Eicosapentaenoic acid; docosahexaenoic acid; docosapentaenoic acid; arachidonic acid; phosphoric acid; 2-furoic acid	0.958	0.915-0.997
Eicosapentaenoic acid; docosahexaenoic acid; docosapentaenoic acid; arachidonic acid; citric acid; *α*-ketoglutaric acid; genistein	0.984	0.962-1.000
Eicosapentaenoic acid; docosahexaenoic acid; docosapentaenoic acid; arachidonic acid; citric acid; *α*-ketoglutaric acid; daidzein	0.984	0.962-1.000
Eicosapentaenoic acid; docosahexaenoic acid; docosapentaenoic acid; arachidonic acid; citric acid; *α*-ketoglutaric acid; phosphoric acid	0.984	0.962-1.000
Eicosapentaenoic acid; docosahexaenoic acid; docosapentaenoic acid; arachidonic acid; citric acid; *α*-ketoglutaric acid; 2-furoic acid	0.984	0.962-1.000
Eicosapentaenoic acid; docosahexaenoic acid; docosapentaenoic acid; arachidonic acid; citric acid; genistein; daidzein	0.957	0.916-0.997
Eicosapentaenoic acid; docosahexaenoic acid; docosapentaenoic acid; arachidonic acid; citric acid; genistein; phosphoric acid	0.958	0.919-0.998
Eicosapentaenoic acid; docosahexaenoic acid; docosapentaenoic acid; arachidonic acid; citric acid; genistein; 2-furoic acid	0.960	0.922-0.998
Eicosapentaenoic acid; docosahexaenoic acid; docosapentaenoic acid; arachidonic acid; citric acid; *α*-ketoglutaric acid; genistein; daidzein	0.983	0.960-1.000
Eicosapentaenoic acid; docosahexaenoic acid; docosapentaenoic acid; arachidonic acid; *α*-ketoglutaric acid; genistein; daidzein; phosphoric acid	0.981	0.957-1.000
Eicosapentaenoic acid; docosahexaenoic acid; docosapentaenoic acid; arachidonic acid; *α*-ketoglutaric acid; genistein; daidzein; 2-furoic acid	0.981	0.957-1.000
Eicosapentaenoic acid; docosahexaenoic acid; docosapentaenoic acid; arachidonic acid; citric acid; *α*-ketoglutaric acid; genistein; daidzein; phosphoric acid	0.984	0.962-1.000
Eicosapentaenoic acid; docosahexaenoic acid; docosapentaenoic acid; arachidonic acid; citric acid; *α*-ketoglutaric acid; genistein; daidzein; 2-furoic acid	0.984	0.962-1.000
Eicosapentaenoic acid; docosahexaenoic acid; docosapentaenoic acid; arachidonic acid; citric acid; *α*-ketoglutaric acid; genistein; daidzein; phosphoric acid; 2-furoic acid	0.984	0.962-1.000

ROC curves were prepared for different metabolite combinations. AUC, area under the curves.

## Data Availability

The data used to support the findings of this study are available from the corresponding authors upon request.

## Supplementary Materials

Supplementary Materials Table S1: other general characteristics of study subjects. We included some clinical data other than Table 1 in the article in this part, including FIB, APTT, PT, TT, HGB, LY#, LY%, MCH, MCHC, MCV, NE#, NE%, P-LCR, PDW, PLT, RBC, WBC, FT3, FT4, TT3, TT4, TSH, A-G, ALB, and albumin; D-BIL, GGT, ID-BIL, T-BIL, TBA, TP, and URIC may be better for us to understand the basic situation of patients. Figure S2: evaluation of system stability throughout the experiment. QC samples are used to determine the status of the instrument and balance chromatography-mass spectrometry system before the sample and to evaluate the stability of the system throughout the experiment. The correlation of QC samples was all close to 1, indicating that the method used has high stability and good data quality. In the PCA analysis diagram, the distribution of QC samples, GDM samples, and control samples is clustered together. These results further indicate that the model we employed is also reliable. Table S3: twenty metabolite molecules with the most significant differences. In this part of the supplementary materials, we selected 20 metabolites with the lowest *p*-value under different models. *p*-value, area under ROC curve, VIP value, and trends are list in the table to make more clearer to the readers. Table S3: the most obvious metabolite on the KEGG pathway. In this part of the supplementary materials, we list the most obvious metabolite on the KEGG pathway, including *p*-value, area under ROC curve, VIP value, and trends. Table S5: the largest metabolites of VIP. In this part of the supplementary materials, we list the largest metabolites of VIP, including *p*-value, area under ROC curve, VIP value, and trends. Figure S6: area under the curves among GDM and controls. This part of the result is the same as that of Table 3. Table 3 shows area under the curves among GDM and controls in a form mode, while Figure S6 shows the picture format.
