# Supplementary material for: Unique Biomarker Characteristics in Gestational Diabetes Mellitus Identified by LC-MS-Based Metabolic Profiling
Source: J Diabetes Res. 2021 Jun 9;2021:6689414. doi: 10.1155/2021/6689414 (PMC8211500; doi:10.1155/2021/6689414)
Supplement: Supplementary Materials — Table S1: other general characteristics of study subjects. We included some clinical data other than Table 1 in the article in this part, including FIB, APTT, PT, TT, HGB, LY#, LY%, MCH, MCHC, MCV, NE#, NE%, P-LCR, PDW, PLT, RBC, WBC, FT3, FT4, TT3, TT4, TSH, A-G, ALB, and albumin; D-BIL, GGT, ID-BIL, T-BIL, TBA, TP, and URIC may be better for us to understand the basic situation of patients. Figure S2: evaluation of system stability throughout the experiment. QC samples are used to determine the status of the instrument and balance chromatography-mass spectrometry system before the sample and to evaluate the stability of the system throughout the experiment. The correlation of QC samples was all close to 1, indicating that the method used has high stability and good data quality. In the PCA analysis diagram, the distribution of QC samples, GDM samples, and control samples is clustered together. These results further indicate that the model we employed is also reliable. Table S3: twenty metabolite molecules with the most significant differences. In this part of the supplementary materials, we selected 20 metabolites with the lowest p-value under different models. p-value, area under ROC curve, VIP value, and trends are list in the table to make more clearer to the readers. Table S3: the most obvious metabolite on the KEGG pathway. In this part of the supplementary materials, we list the most obvious metabolite on the KEGG pathway, including p-value, area under ROC curve, VIP value, and trends. Table S5: the largest metabolites of VIP. In this part of the supplementary materials, we list the largest metabolites of VIP, including p-value, area under ROC curve, VIP value, and trends. Figure S6: area under the curves among GDM and controls. This part of the result is the same as that of Table 3. Table 3 shows area under the curves among GDM and controls in a form mode, while Figure S6 shows the picture format. [file 6689414.f1.doc]

**Supplementary Materials**

**Supplementary T**able 1. Other general characteristics of study subjects.

| Characteristic | No GDM | GDM | *p* value^a^ |
| --- | --- | --- | --- |
| FIB (g/L) | 4.25±0.64 | 4.64±0.75 | 0.028 |
| APTT (s) | 31.87±2.99 | 32.26±5.57 | 0.724 |
| PT (s) | 12.38±0.56 | 12.62±0.45 | 0.053 |
| TT (s) | 14.64±0.61 | 14.78±0.47 | 0.290 |
| HGB (g/L) | 116.06±10.89 | 117.21±9.73 | 0.648 |
| LY# (10^9/L) | 1.91±1.01 | 1.75±0.47 | 0.405 |
| LY%（%） | 18.72±4.37 | 17.21±4.44 | 0.163 |
| MCH (pg） | 31.43±1.66 | 30.74±2.62 | 0.199 |
| MCHC (g/L) | 332.03±9.10 | 336.85±11.07 | 0.054 |
| MCV (fl） | 94.65±4.17 | 90.31±7.69 | 0.005 |
| NE# (10^9/L) | 7.10±1.87 | 7.77±1.76 | 0.133 |
| NE% (%) | 74.03±4.77 | 72.93±12.57 | 0.631 |
| P-LCR (%) | 27.70±6.54 | 26.30±7.52 | 0.416 |
| PDW (fl) | 11.83±1.61 | 11.65±2.21 | 0.708 |
| PLT (10^9/L) | 224.15±52.09 | 237.09±50.46 | 0.302 |
| RBC (10^12/L) | 3.70±0.39 | 3.83±0.35 | 0.153 |
| WBC (10^9/L) | 9.56±2.27 | 10.36±2.06 | 0.129 |
| FT3 (pmol/L) | 3.86±0.36 | 4.14±0.50 | 0.008 |
| FT4 (pmol/L) | 11.06±1.24 | 10.64±1.08 | 0.143 |
| TT3 (pmol/L) | 1.86±0.29 | 2.11±0.42 | 0.004 |
| TT4 (pmol/L) | 130.30±18.5 | 124.28±20.74 | 0.211 |
| TSH (mIU/L) | 1.77±0.77 | 1.65±0.94 | 0.595 |
| A-G | 1.31±0.13 | 1.29±0.14 | 0.512 |
| ALB (g/L) | 31.49±1.92 | 36.10±1.85 | 0.402 |
| D-BIL (μmol/L) | 2.24±0.87 | 2.25±0.97 | 0.990 |
| GGT (U/L) | 11.21±4.2 | 13.53±6.78 | 0.094 |
| ID-BIL (μmol/L) | 4.76±2.15 | 4.59±2.08 | 0.745 |
| T-BIL (μmol/L) | 7.01±3.09 | 6.84±2.81 | 0.813 |
| TBA (μmol/L) | 2.15±1.48 | 2.24±1.21 | 0.788 |
| TP (g/L) | 64.48±3.91 | 64.32±3.37 | 0.871 |
| URIC (μmol/L) | 230.85±40.67 | 223.88±37.45 | 0.465 |

**p* < 0.05, compared with control group. FIB, Fibrinogen; APTT, activated partial thromboplastin time; PT, prothrombin time;TT, thrombin time; HGB, hemoglobin concentration; LY#, Lymphocyte absolute value; LY%, Percentage of lymphocytes; MCH, Mean Corpuscular Hemoglobin; MCHC, mean corpuscular hemoglobin concentration; MCV, mean corpuscular volume; NE#, absolute neutrophil count; NE%, neutrophil percentage; P-LCR, platelet large cell ratio; PDW, platelet distribution width; PLT, platelet; RBC, red blood cell; WBC, white blood cell; FT3, Free triiodothyronine; FT4, free thyroxine; TT3, total triiodothyronine; TT4, total thyroxine; TSH, High sensitivity thyrotrophin; A-G, albumin globulin ratio; ALB, albumin; D-BIL, direct bilirubin; GGT, gamma-glutamyltransferase; ID-BIL, indirect bilirubin; T-BIL, total bilirubin; TBA, Total bile acid; TP, total protein; URIC, Uric acid; SD, standard deviation.





**Supplementary Figure 1 Evaluation of system stability throughout the experiment.** QC samples are used to determine the status of the instrument and balance chromatography-mass spectrometry system before the sample, and to evaluate the stability of the system throughout the experiment. a,c,e. positive ion mode; b.d.f negative ion mode.

**Supplementary Table 2. Twenty metabolite molecules with the most significant differences.**

| ID | Name_des | *P* value | ROC | VIP | Up/Down |
| --- | --- | --- | --- | --- | --- |
| Com_26725_pos | 3,3'-[8-(1-Hydroxy-5,9,13-trimethyl-4,8,12-tetradecatrien-1-yl)-3,7,12,17-tetramethyl-13-vinyl-1,2,3,6,7,8,9,12,17,18,22,24-dodecahydroporphyrin-2,18-diyl]dipropanoic acid | 5.93E-14 | 0.96 | 2.74 | down |
| Com_7730_pos | 2-arachidonoylglycerol | 6.84E-14 | 0.95 | 1.80 | up |
| Com_6594_pos | (1Z,2S)-N-[(2S,3R,4E,8E)-1,3-Dihydroxy-4,8-octadecadien-2-yl]-2-hydroxypentadecanimidic acid | 1.58E-13 | 0.93 | 3.46 | down |
| Com_7090_pos | 1-Glyceryl stearate | 4.33E-12 | 0.93 | 1.83 | up |
| Com_5721_pos | D-Glucono-delta-lactone | 9.75E-12 | 0.95 | 1.29 | up |
| Com_20183_pos | 3-(Icosanoyloxy)-4-(trimethylammonio)butanoate | 1.18E-11 | 0.92 | 2.01 | down |
| Com_10190_pos | 3-Aminopropane-1-sulfonic acid | 3.03E-11 | 0.90 | 2.80 | down |
| Com_4694_pos | S-3-oxodecanoyl cysteamine | 4.78E-11 | 0.90 | 2.78 | down |
| Com_2150_pos | (1R,2S,5aR,5bR,7aS,10R,12bR)-2-Hydroxy-10-isopropenyl-3,3,5a,5b,12b-pentamethyloctadecahydrodicyclopenta[a,i]phenanthrene-1,7a(1H)-dicarboxylic acid | 7.42E-11 | 0.90 | 1.57 | up |
| Com_7931_pos | Isopropyl unoprostone | 8.57E-11 | 0.91 | 1.93 | up |
| Com_13100_pos | (1Z,2S)-N-[(2S,3R,4E,8E)-1,3-Dihydroxy-4,8-octadecadien-2-yl]-2-hydroxytridecanimidic acid | 1.73E-10 | 0.90 | 2.31 | down |
| Com_19946_pos | O-behenoylcarnitine | 1.73E-10 | 0.89 | 2.74 | down |
| Com_9510_pos | 3,3'-[10,15-Bis(1-{[2-amino-3-(methylamino)-3-oxopropyl]sulfanyl}ethyl)-14-ethyl-5,9,19-trimethyl-21,22,23,24-tetraazapentacyclo[16.2.1.1~3,6~.1~8,11~.1~13,16~]tetracosa-1,3,5,7,9,11,13(22),14,17,19-decaene-4,20-diyl]dipropanoic acid | 2.55E-10 | 0.89 | 1.33 | up |
| Com_18749_pos | Biocytin | 3.26E-10 | 0.83 | 3.21 | up |
| Com_15885_pos | 5beta-scymnol | 3.77E-10 | 0.90 | 1.38 | up |
| Com_10833_pos | 1-stearoyl-2-arachidonoyl-sn-glycero-3-phosphoserine | 5.19E-10 | 0.91 | 1.40 | up |
| Com_15361_pos | androstenol | 5.37E-10 | 0.91 | 1.20 | up |
| Com_14081_pos | 2,3-Bis[(9Z,12Z)-9,12-octadecadienoyloxy]propyl (8Z,11Z,14Z)-8,11,14-icosatrienoate | 5.47E-10 | 0.84 | 6.49 | up |
| Com_5983_pos | DINP | 6.78E-10 | 0.89 | 1.42 | up |
| Com_13502_pos | 2638 | 8.17E-10 | 0.88 | 1.42 | up |
| Com_4315_neg | 12-HSA | 2.73E-15 | 0.99 | 3.10 | down |
| Com_9021_neg | (1S,2R,5S)-2-Isopropyl-5-methylcyclohexyl 3-oxobutanoate | 1.61E-13 | 0.93 | 3.24 | up |
| Com_9154_neg | (Z)-N-[(2R)-2-Amino-1-hydroxy-3-{[(1E)-N-hydroxy-8-(methylsulfanyl)octanimidoyl]sulfanyl}propylidene]glycine | 5.37E-12 | 0.92 | 2.32 | down |
| Com_7453_neg | Adhyperforin | 2.20E-11 | 0.91 | 2.17 | down |
| Com_4200_neg | Tazarotene | 9.42E-11 | 0.90 | 2.82 | down |
| Com_418_neg | Typhasterol | 5.34E-10 | 0.91 | 2.09 | down |
| Com_108_neg | Citric acid | 1.02E-09 | 0.84 | 3.43 | up |
| Com_4310_neg | Quinalphos | 1.49E-09 | 0.88 | 5.73 | down |
| Com_5332_neg | alpha-Ketoglutaric acid | 2.17E-09 | 0.88 | 2.00 | up |
| Com_14848_neg | Tin(II) fluoride | 2.34E-09 | 0.87 | 1.68 | up |
| Com_7752_neg | Clozapine | 3.47E-09 | 0.90 | 2.08 | up |
| Com_8048_neg | D-Xylonic acid | 1.00E-08 | 0.87 | 1.84 | down |
| Com_740_neg | Phosphoric acid | 1.42E-08 | 0.84 | 4.31 | up |
| Com_12132_neg | Leukotriene B4 Ethanolamide | 1.46E-08 | 0.85 | 2.04 | up |
| Com_8273_neg | pyriproxyfen | 1.89E-08 | 0.86 | 1.84 | down |
| Com_10209_neg | gamma-Glutamyl-gamma-glutamylglutamic acid | 2.17E-08 | 0.91 | 2.14 | up |
| Com_8878_neg | Tricosylic acid | 2.50E-08 | 0.89 | 2.35 | down |
| Com_7683_neg | 2,6-di-tert-butyl-4-ethylphenol | 3.24E-08 | 0.90 | 3.36 | down |
| Com_3685_neg | Malonic acid | 3.64E-08 | 0.94 | 2.37 | up |
| Com_6856_neg | (8R,9S)-fumigaclavine C | 4.66E-08 | 0.88 | 2.86 | up |

FC, Fold change (GDM case/control). VIP, Variable Importance in the Projection. ROC, Receiver Operating Characteristic curve.

**Supplementary Table 3. The most obvious metabolite on the KEGG pathway.**

| **ID** | **Name_des** | *P***-value** | **ROC** | **VIP** | **Up/Down** |
| --- | --- | --- | --- | --- | --- |
| Biosynthesis of unsaturated fatty acids |  |  |  |  |  |
| Com_962_pos | Eicosapentaenoic acid | 4.95E-03 | 0.69 | 2.28 | down |
| Com_384_pos | Docosahexaenoic acid | 1.62E-06 | 0.83 | 1.60 | up |
| Com_2412_pos | Docosapentaenoic acid | 6.97E-09 | 0.88 | 2.23 | up |
| Com_1075_pos | Arachidonic acid | | 6.97E-09 | | --- | | 3.85E-06 | | 0.80 | 1.06 | up |
| Biosynthesis of phenylpropanoids |  |  |  |  |  |
| Com_108_neg | Citric acid | 1.02E-09 | 0.84 | 3.43 | up |
| Com_10984_neg | Isoliquiritigenin | 9.28E-03 | 0.67 | 2.11 | down |
| Com_5332_neg | alpha-Ketoglutaric acid | 2.17E-09 | 0.88 | 2.00 | up |
| Com_7586_neg | Genistein | 6.15E-03 | 0.75 | 1.17 | down |
| Com_8251_neg | Daidzein | 4.12E-02 | 0.62 | 1.50 | down |
| Carbon fixation pathways in prokaryotes |  |  |  |  |  |
| Com_108_neg | Citric acid | 1.02E-09 | 0.84 | 3.43 | up |
| Com_5332_neg | alpha-Ketoglutaric acid | 2.17E-09 | 0.88 | 2.00 | up |
| Biosynthesis of terpenoids and steroids |  |  |  |  |  |
| Com_108_neg | Citric acid | 1.02E-09 | 0.84 | 3.43 | up |
| Com_5332_neg | alpha-Ketoglutaric acid | 2.17E-09 | 0.88 | 2.00 | up |
| Two-component system |  |  |  |  |  |
| Com_108_neg | Citric acid | 1.02E-09 | 0.84 | 3.43 | up |
| Com_740_neg | Phosphoric acid | 1.42E-08 | 0.84 | 4.31 | up |
| Ascorbate and aldarate metabolism |  |  |  |  |  |
| Com_3499_neg | DEHYDROASCORBIC ACID | 1.80E-04 | 0.75 | 1.40 | up |
| Com_5332_neg | alpha-Ketoglutaric acid | 2.17E-09 | 0.88 | 2.00 | up |
| Furfural degradation |  |  |  |  |  |
| Com_5332_neg | alpha-Ketoglutaric acid | 2.17E-09 | 0.88 | 2.00 | up |
| Com_783_neg | 2-Furoic acid | 1.97E-06 | 0.81 | 3.15 | up |
| Isoflavonoid biosynthesis |  |  |  |  |  |
| Com_7586_neg | Genistein | 6.15E-03 | 0.75 | 1.17 | down |
| Com_8251_neg | Daidzein | 4.12E-02 | 0.62 | 1.50 | down |
| Biosynthesis of alkaloids derived from shikimate pathway |  |  |  |  |  |
| Com_108_neg | Citric acid | 1.02E-09 | 0.84 | 3.43 | up |
| Com_12278_neg | Cephaeline | 1.25E-05 | 0.80 | 1.86 | up |
| Com_5332_neg | alpha-Ketoglutaric acid | 2.17E-09 | 0.88 | 2.00 | up |
| Biosynthesis of secondary metabolites |  |  |  |  |  |
| Com_108_neg | Citric acid | 1.02E-09 | 0.84 | 3.43 | up |
| Com_10984_neg | Isoliquiritigenin | 9.28E-03 | 0.67 | 2.11 | down |
| Com_12278_neg | Cephaeline | 1.25E-05 | 0.80 | 1.86 | up |
| Com_1834_neg | Methyl Jasmonate | 7.25E-03 | 0.70 | 1.56 | up |
| Com_418_neg | Typhasterol | 5.34E-10 | 0.91 | 2.09 | down |
| Com_7586_neg | Genistein | 6.15E-03 | 0.75 | 1.17 | down |
| Com_8251_neg | Daidzein | 4.12E-02 | 0.62 | 1.50 | down |

FC, Fold change (GDM case/control). VIP, Variable Importance in the Projection. ROC, Receiver Operating Characteristic curve.

**Supplementary Table 4. The largest metabolites of VIP.**

| **ID** | **Name_des** | ***P-value*** | **ROC** | **VIP** | **Up/Down** |
| --- | --- | --- | --- | --- | --- |
| Com_14081_pos | 2,3-Bis[(9Z,12Z)-9,12-octadecadienoyloxy]propyl (8Z,11Z,14Z)-8,11,14-icosatrienoate | 5.47E-10 | 0.84 | 6.49 | up |
| Com_8924_pos | 2-Acetamido-4-O-(2-acetamido-2-deoxy-beta-D-glucopyranosyl)-2-deoxy-1-O-{hydroxy[(hydroxy{[(6E)-3,7,11-trimethyl-6,10-dodecadien-1-yl]oxy}phosphoryl)oxy]phosphoryl}-beta-D-glucopyranose | 6.07E-08 | 0.79 | 5.49 | up |
| Com_5615_pos | PC(o-22:0/18:3(6Z,9Z,12Z)) | 7.89E-08 | 0.76 | 5.32 | up |
| Com_8011_pos | 1,3-distearoyl-2-oleoylglycerol | 3.66E-08 | 0.79 | 4.53 | up |
| Com_1811_pos | 4-Iodoanisole | 3.54E-07 | 0.75 | 4.29 | up |
| Com_22394_pos | 1-(Tetradecanoyloxy)-3-[(9Z)-9-tetradecenoyloxy]-2-propanyl pentadecanoate | 3.68E-07 | 0.84 | 4.23 | up |
| Com_9648_pos | 2-methoxy-6-(all-trans-nonaprenyl)phenol | 2.34E-07 | 0.80 | 4.20 | up |
| Com_7944_pos | (2S,3R)-3-Hydroxy-2-[(15Z)-15-tetracosenoylamino]octadecyl 5-acetamido-6-[(1S,2R)-2-({5-acetamido-3,5-dideoxy-6-[(1R,2R)-1,2,3-trihydroxypropyl]-beta-L-threo-hex-2-ulopyranonosyl}oxy)-1,3-dihydroxypropyl]-3,5-dideoxy-beta-L-threo-hex-2-ulopyranonosyl-(2->3)-[2-deoxy-2-(2-oxopropyl)-beta-D-galactopyranosyl-(1->4)]-beta-D-galactopyranosyl-(1->4)-beta-D-glucopyranoside | 6.82E-07 | 0.77 | 4.18 | up |
| Com_13243_pos | O-Sulfotyrosylisoleucyl-O-sulfotyrosylthreonine | 8.74E-07 | 0.77 | 3.84 | up |
| Com_16258_pos | 7,9-Dibromo-N-{3-[2,6-dibromo-4-(2-oxo-1,3-oxazolidin-5-yl)phenoxy]-2-hydroxypropyl}-10-hydroxy-8-methoxy-1-oxa-2-azaspiro[4.5]deca-2,6,8-triene-3-carboxamide | 8.33E-09 | 0.82 | 3.69 | up |
| Com_10073_pos | Glucosylceramide (d18:1/22:0) | 1.90E-07 | 0.78 | 3.66 | up |
| Com_19561_pos | PG(18:2(9Z,12Z)/18:2(9Z,12Z)) | 4.26E-07 | 0.82 | 3.57 | up |
| Com_6594_pos | (1Z,2S)-N-[(2S,3R,4E,8E)-1,3-Dihydroxy-4,8-octadecadien-2-yl]-2-hydroxypentadecanimidic acid | 1.58E-13 | 0.93 | 3.46 | down |
| Com_3558_pos | (2S)-1-Hydroxy-3-[(5Z,8Z,11Z,14Z,17Z)-5,8,11,14,17-icosapentaenoyloxy]-2-propanyl (4Z,7Z,10Z,13Z,16Z,19Z)-4,7,10,13,16,19-docosahexaenoate | 1.86E-08 | 0.82 | 3.45 | up |
| Com_2683_pos | Methyl 5-{(3aS,5R,6R,6aS)-5-hydroxy-6-[(1E,3R)-3-hydroxy-1-octen-1-yl]-1,3a,4,5,6,6a-hexahydro-2-pentalenyl}pentanoate | 1.63E-03 | 0.84 | 3.30 | up |
| Com_13727_pos | Methyl (2S,2aS,3a'R,4a'R,5aR,12bR,12b'R,12c'R,15aR,15a'S,17a'S)-12c'-hydroxy-10'-methoxy-2',3',4,4a',5,6,8,12c',13,13',14,14',17',17a'-tetradecahydro-4'H,5'H,16'H-spiro[furo[2',3':7,8]indolizino[8,1-cd]carbazole-2,6'-furo[2',3':7,8]indolizino[8,1-cd]pyrido[1,2,3-lm]carbazole]-7-carboxylate | 2.47E-09 | 0.83 | 3.30 | up |
| Com_530_pos | O-heptanoylcarnitine | 1.09E-04 | 0.78 | 3.25 | down |
| Com_8742_pos | 2033 | 1.03E-08 | 0.83 | 3.24 | down |
| Com_18749_pos | Biocytin | 3.26E-10 | 0.83 | 3.21 | up |
| Com_4585_pos | 11-Ketotestosterone | 1.48E-06 | 0.82 | 3.10 | down |
| Com_1199_neg | 5beta-cholanoic acid | 7.52E-05 | 0.81 | 6.82 | down |
| Com_4310_neg | Quinalphos | 1.49E-09 | 0.88 | 5.73 | down |
| Com_740_neg | Phosphoric acid | 1.42E-08 | 0.84 | 4.31 | up |
| Com_1624_neg | Nervonic acid | 8.90E-08 | 0.95 | 4.13 | down |
| Com_1824_neg | 12(S)-HHT | 5.46E-07 | 0.83 | 3.97 | down |
| Com_2279_neg | L-alpha-lysophosphatidylcholine | 3.05E-06 | 0.80 | 3.92 | down |
| Com_91_neg | Retinyl acetate | 3.11E-04 | 0.80 | 3.91 | down |
| Com_4739_neg | 1730452 | 8.52E-05 | 0.80 | 3.79 | down |
| Com_8573_neg | UNII:Z5JO63XGNK | 3.89E-07 | 0.85 | 3.57 | down |
| Com_12979_neg | Dehydroglycine | 2.58E-06 | 0.84 | 3.46 | down |
| Com_108_neg | Citric acid | 1.02E-09 | 0.84 | 3.43 | up |
| Com_7683_neg | 2,6-di-tert-butyl-4-ethylphenol | 3.24E-08 | 0.90 | 3.36 | down |
| Com_9021_neg | (1S,2R,5S)-2-Isopropyl-5-methylcyclohexyl 3-oxobutanoate | 1.61E-13 | 0.93 | 3.24 | up |
| Com_1929_neg | 1,3-Dihydroxy-2-propanyl tetracosanoate | 2.73E-03 | 0.80 | 3.19 | down |
| Com_783_neg | 2-Furoic acid | 1.97E-06 | 0.81 | 3.15 | up |
| Com_4315_neg | 12-HSA | 2.73E-15 | 0.99 | 3.10 | down |
| Com_943_neg | 11,12-Epoxy-(5Z,8Z,11Z)-icosatrienoic acid | 4.48E-04 | 0.74 | 3.09 | down |
| Com_745_neg | Citraconic acid | 7.53E-08 | 0.80 | 2.98 | up |
| Com_11661_neg | N-[3-Carboxy-2-(carboxymethyl)-2-hydroxypropanoyl]glutamic acid | 1.62E-07 | 0.80 | 2.92 | up |
| Com_3112_neg | Desoxycorticosterone acetate | 3.44E-04 | 0.73 | 2.88 | down |

FC, Fold change (GDM case/control). VIP, Variable Importance in the Projection. ROC, Receiver Operating Characteristic curve.

**
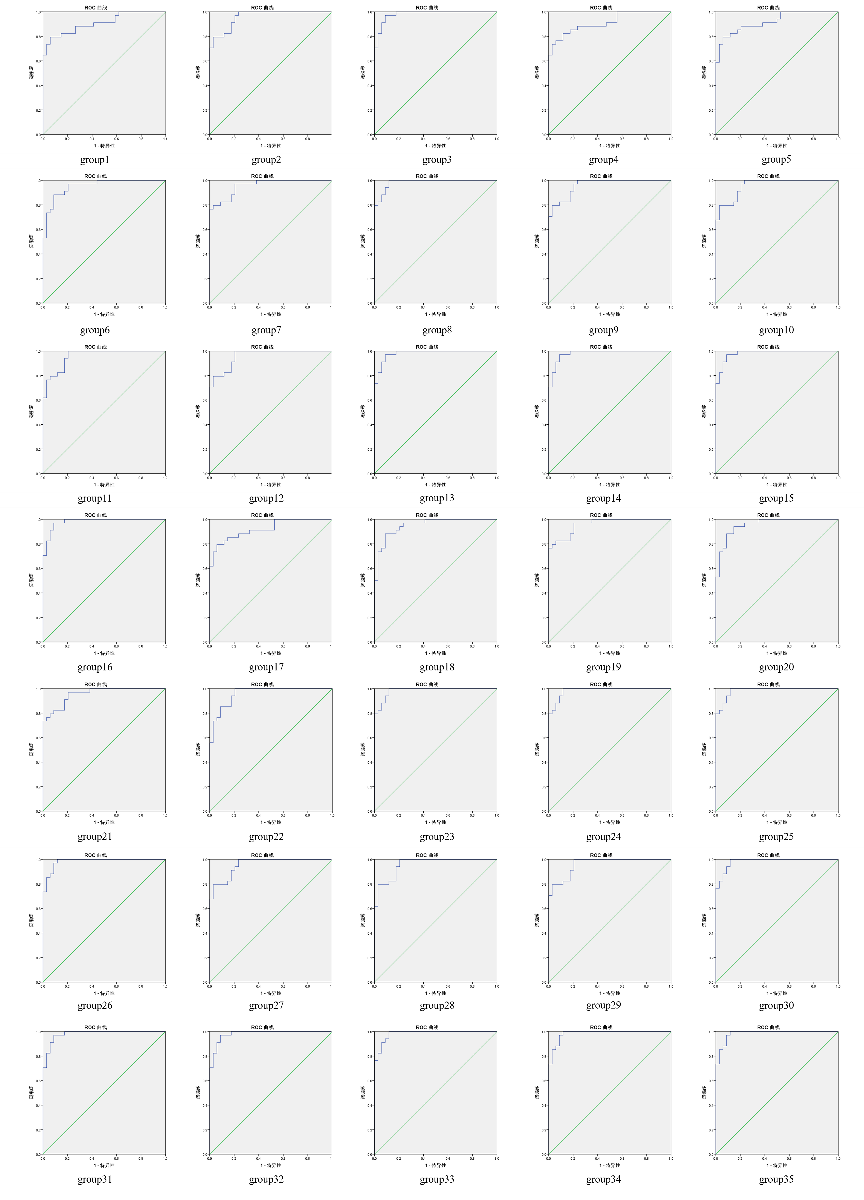
**

**Supplementary Figure 2 Area under the curves among GDM and control.** ROC curves were prepared for different metabolite combinations. AUC, Area under the curves.
